# RNA degradation compromises the reliability of microRNA expression profiling

**DOI:** 10.1186/1472-6750-9-102

**Published:** 2009-12-21

**Authors:** David Ibberson, Vladimir Benes, Martina U Muckenthaler, Mirco Castoldi

**Affiliations:** 1 Genomics Core Facility, EMBL, Meyerhofstraße 1 D-69117 Heidelberg, Germany; 2 Department of Pediatric Oncology, Hematology and Immunology, University of Heidelberg, Im Neuenheimer Feld 156, D-69120, Heidelberg, Germany; 3 Molecular Medicine Partnership Unit, Im Neuenheimer Feld 156, D-69120, Heidelberg, Germany

## Abstract

**Background:**

MicroRNAs are small non-coding RNAs that post-transcriptionally regulate gene expression and their expression is frequently altered in human diseases, including cancer. To correlate clinically relevant parameters with microRNA expression, total RNA is frequently prepared from samples that were archived for various time periods in frozen tissue banks but, unfortunately, RNA integrity is not always preserved in these frozen tissues. Here, we investigate whether experimentally induced RNA degradation affects microRNA expression profiles.

**Results:**

Tissue samples were maintained on ice for defined time periods prior to total RNA extraction, which resulted in different degrees of RNA degradation. MicroRNA expression was then analyzed by microarray analysis (miCHIP) or microRNA-specific real-time quantitative PCR (miQPCR). Our results demonstrate that the loss of RNA integrity leads to in unpredictability of microRNA expression profiles for both, array-based and miQPCR assays.

**Conclusion:**

MicroRNA expression cannot be reliably profiled in degraded total RNA. For the profiling of microRNAs we recommend use of RNA samples with a RNA integrity number equal to or above seven.

## Background

MicroRNAs (miRNAs) are evolutionary conserved, small, non-coding RNAs that post-transcriptionally control the expression of protein coding genes [1,2] in diverse cellular processes such as differentiation [3,4], proliferation [5] and apoptosis [6]. Differential miRNA expression has been detected in human malignancies [7-10] and thus, specific changes in miRNA expression may provide valuable diagnostic and prognostic information [11,12]. The ability to reliably quantify miRNA expression in archived tissue collections would simplify retrospective studies by allowing correlations between a given disease state with a specific miRNA signature. Large numbers of clinical samples are preserved either in liquid nitrogen (preserving nucleic acid and proteins) or as Formalin-Fixed Paraffin-Embedded blocks (FFPE, preserving tissue morphology). For these samples, the clinical history and the clinical outcome (e.g. baseline parameters, response to treatment, and recurrence of the disease or survival rate) are frequently known. Unfortunately, RNA integrity is not always preserved in these archived tissues, with studies indicating that RNA extracted from FFPE blocks is particularly highly degraded [13]. However, the miRNA profiles obtained from FFPE extracted and snap-frozen extracted total RNA are comparable [14-16], suggesting that miRNAs may escape the chemical degradation induced by formalin fixation.

To our knowledge, RNA integrity and its effect on miRNA detection has not been studied in snap-frozen specimens. We therefore addressed the question to what extent total RNA degradation affects miRNA profiles. Our data show that total RNA degradation in defrosted tissues significantly affects miRNA integrity. The lower the quality of total RNA, the higher the proportion of miRNAs that show aberrant signal intensities in microarray and qPCR based approaches. Importantly, we could not identify any systematic effect on the degradation of specific miRNAs that would allow for appropriate correction. Based on these findings, we concluded that reliable miRNA expression profiles using microarrays and qPCR are only achieved if total RNA with RNA Integrity Number (RIN) equal to or above seven is used.

## Results

### RNA degradation in archived snap-frozen tissues

**Figure 1:**
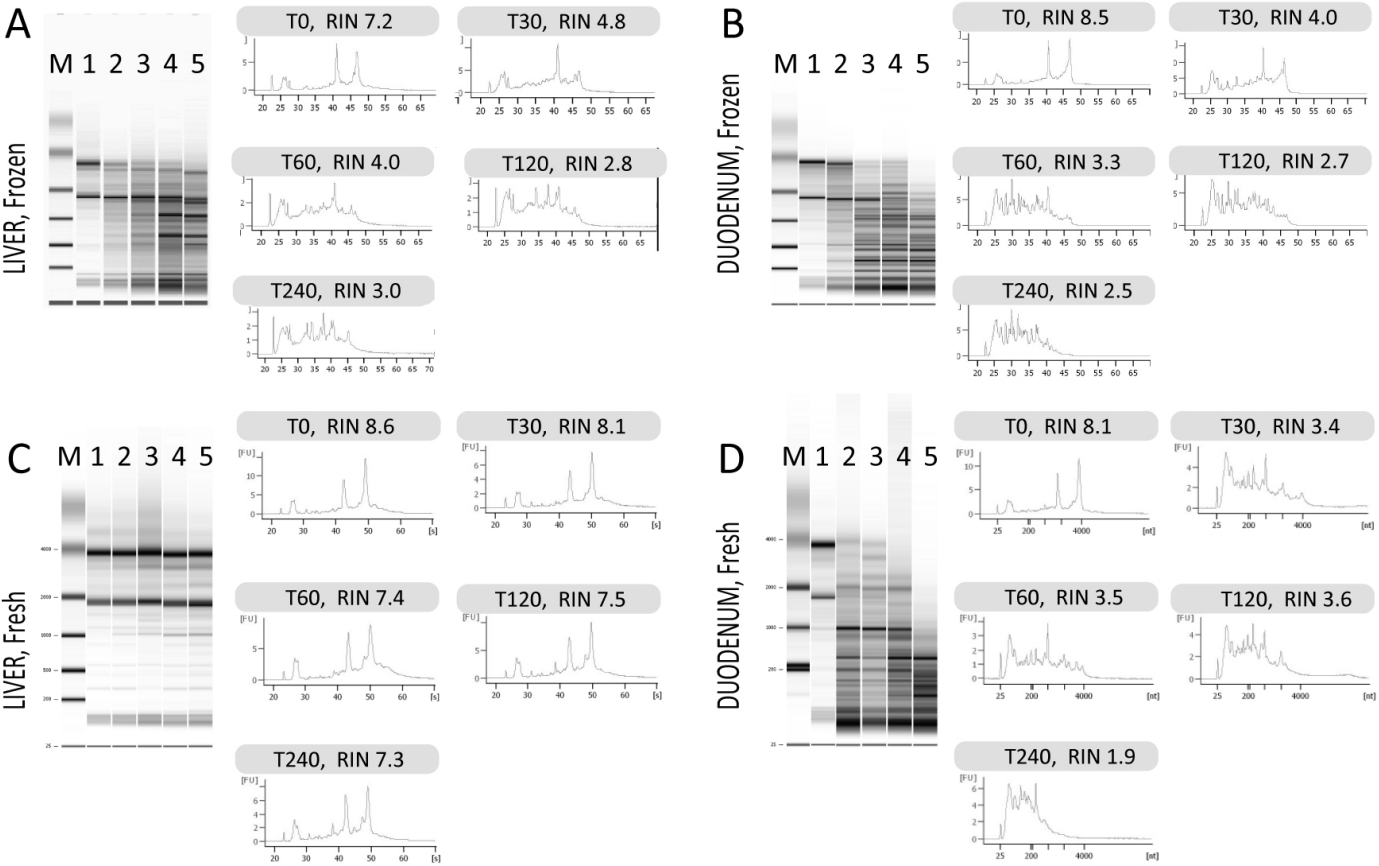
**Assessment of total RNA degradation**. Electropherograms from high quality total RNA present three distinct peaks (28S, 18 S and 5S). The presences of multiple peaks or smears indicate RNA degradation. Electropherograms of total RNA extracted from snap-frozen liver (A) or duodenal (B) samples or from total RNA extracted from fresh liver(C) or fresh duodenum (D) immediately (T0) or after incubation in ice for 30 min (T30), 60 min (T60), 120 min (T120) and 240 min (T240). Total RNA extracted from snap-frozen tissues and fresh duodenum is affected by degradation as early as 30 min on ice. RNA extracted from fresh livers is only slightly affected by degradation even after 4 hours in ice. (M, Marker; 1, T0; 2, T30; 3, T60; 4, T120 and 5, T240).

RNA can be readily damaged by heat, UV, acid or base catalyzed hydrolysis and by enzymatic degradation. However, individually these approaches do not replicate the diverse insults that occur during the extraction of total RNA from frozen tissues. To mimic total RNA degradation in snap-frozen livers and duodenums from mice, tissues were stored in a -80°C freezer and then transferred to dry ice. Each frozen tissue was sliced into five identical pieces which then were transferred into eppendorf tubes. At time point zero (T0), all samples were placed on ice and total RNA was extracted immediately from (T0) and at later time points [30 min (T30), 60 min (T60), 120 min (T120) and 240 min (T240)]. RNA integrities were assessed using the Agilent Bioanalyzer 2100 which calculates RIN values of assayed RNAs (Figure 1). The RIN is the computational output of an algorithm which ranks several parameters obtained from the electropherograms, assigning a numerical value to RNA integrity [17,18]. We found that at T0, the RNA integrity for both tissues was above 7, indicating good quality total RNA. However, 30 min on ice was sufficient to reduce RNA integrity, indicated by the decrease in RINs (Figure 1A, liver; 1B, duodenum). These findings indicate that RNA degradation can take place in defrosted tissue at low temperature (i.e. on ice).

### RNA degradation in freshly harvested tissues

For comparison, we assessed the extent to which RNA integrity is preserved in freshly harvested tissues when tissue processing is delayed. Liver and duodenum samples were collected from mice and either processed immediately or maintained on ice, as described above. Bioanalyzer electropherograms of the liver samples did not indicate any significant total RNA degradation even when samples remained on ice for up to 4 hrs (Figure 1C). By contrast, duodenal samples (which are rich in RNases) do show high susceptibility to degradation similar to the snap-frozen defrosted material (Figure 1D). These findings suggest that tissues such as duodenum or pancreas should either be processed immediately or snap-frozen and processed individually.

### miRNAs are degraded in low integrity total RNA

**Figure 2:**
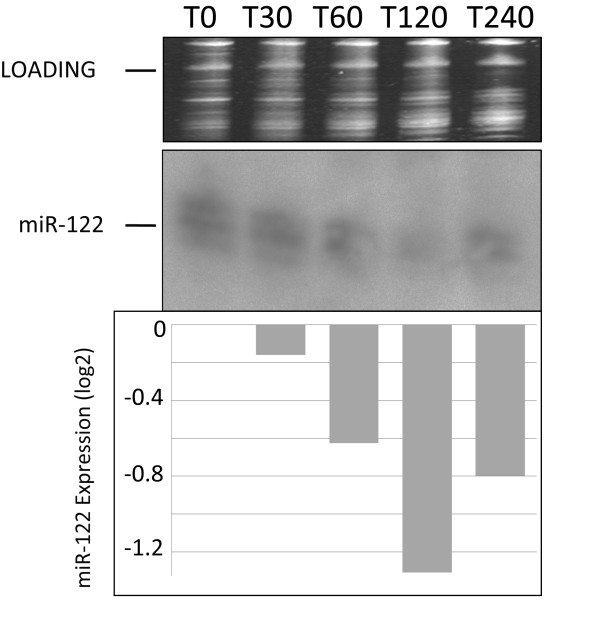
**Analysis of miRNA degradation by Northern blot analysis**. To evaluate how degradation of total RNA affects miRNA integrity the liver specific miR-122 was selected. The top panel shows ethidium bromide stained 5S RNA as a loading control. In the bottom panel background-corrected signal intensities of the northern blot are calculated by ImageJ. The signal corresponding to the miRNA with the highest signal intensity (T0) was set to 100%, and signals for the other samples were calculated as a percentage of it. The position of the mature miR-122 is indicated (middle panel).

A commonly held belief is that small RNAs, including miRNAs, are only marginally affected by degradation, compared to larger mRNAs. To evaluate if miRNA integrity is affected by total RNA degradation, we analyzed miR-122 expression in previously frozen hepatic samples (Figure 2) by northern blotting. Northern blotting offers both quantitative and qualitative information about individual miRNAs [19]. Visual inspection of the bands and phosphoimager quantitation of the signals clearly show a decrease in miR-122 intensity in those RNA samples with lower RIN values. By design, all analyzed RNAs were prepared from the same liver and in consequence differences in miR-122 signals are attributable to RNA degradation. Total RNA samples with the lowest RIN values exhibit the lowest miR-122 signals. For example, following 120 min incubation on ice, total RNA prepared from liver shows a RIN value of 2.8 and has 2.6-fold less miR-122 signal compared to T0 control. Based on this result, we predict that miRNAs are indeed susceptible to degradation and that miRNAs within degraded samples are affected by the degradation process.

### miRNA expression profiling of degraded RNAs

**Figure 3:**
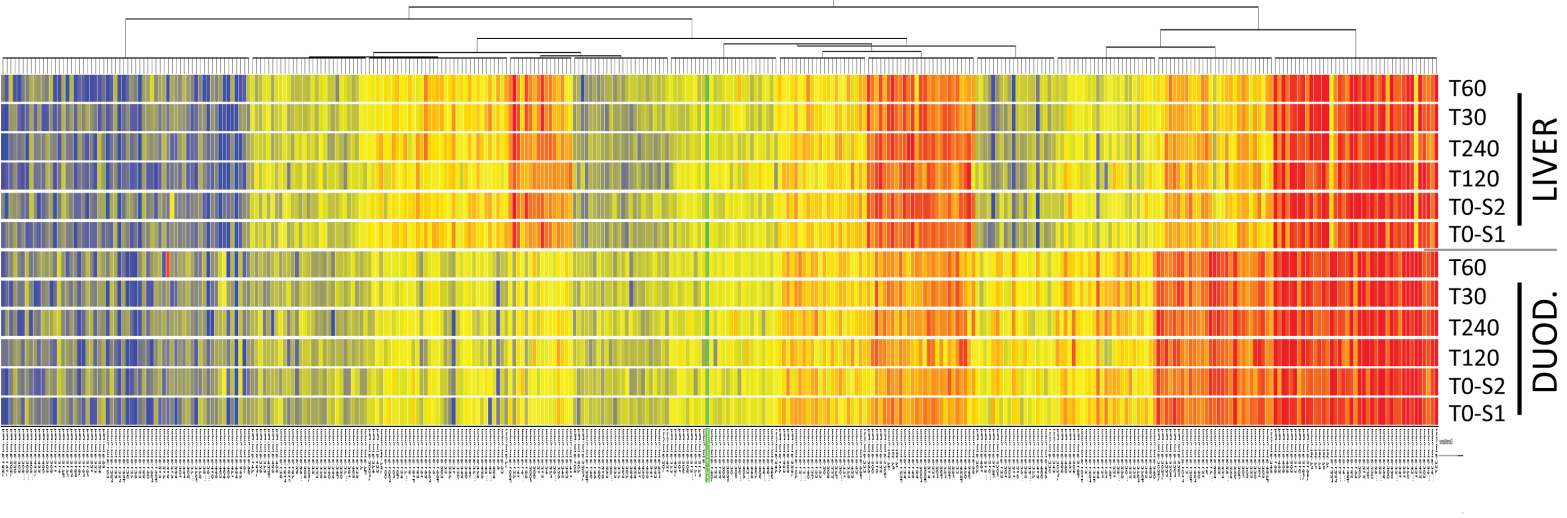
**miCHIP analysis of miRNA expression**. Hierarchical clustering was used to assess which samples cluster together based on their miRNA expression profiles. The data show that even severely degraded total RNA samples are defined by tissue-specific miRNA signatures. The key color bar shows miRNA expression levels (dark red indicates high signal intensities, while dark blue indicates no detectable signal).

The introduction of microarray technology has allowed the analysis of the complete transcriptome of a given cell-type or tissue in a single experiment. We have recently established a novel microarray platform (miCHIP) that accurately and sensitively monitors the genome-wide expression of known mature miRNAs and that discriminates between closely related miRNA family members [20,21]. To assess to which extent total RNA degradation affects miRNA expression profiles, we hybridized total RNAs extracted from liver and duodenum to the miCHIP microarray. Hierarchical clustering (HCL), by Pearson correlation, was used to cluster samples based on their miRNA expression profiles (Figure 3). These analyses show that even samples with the most degraded RNAs still preserve a tissue-specific miRNA signature. Based solely upon miCHIP array data analysis with HCL, we can conclude that RNA degradation does not significantly compromise miRNA tissue-specific signatures.

**Figure 4:**
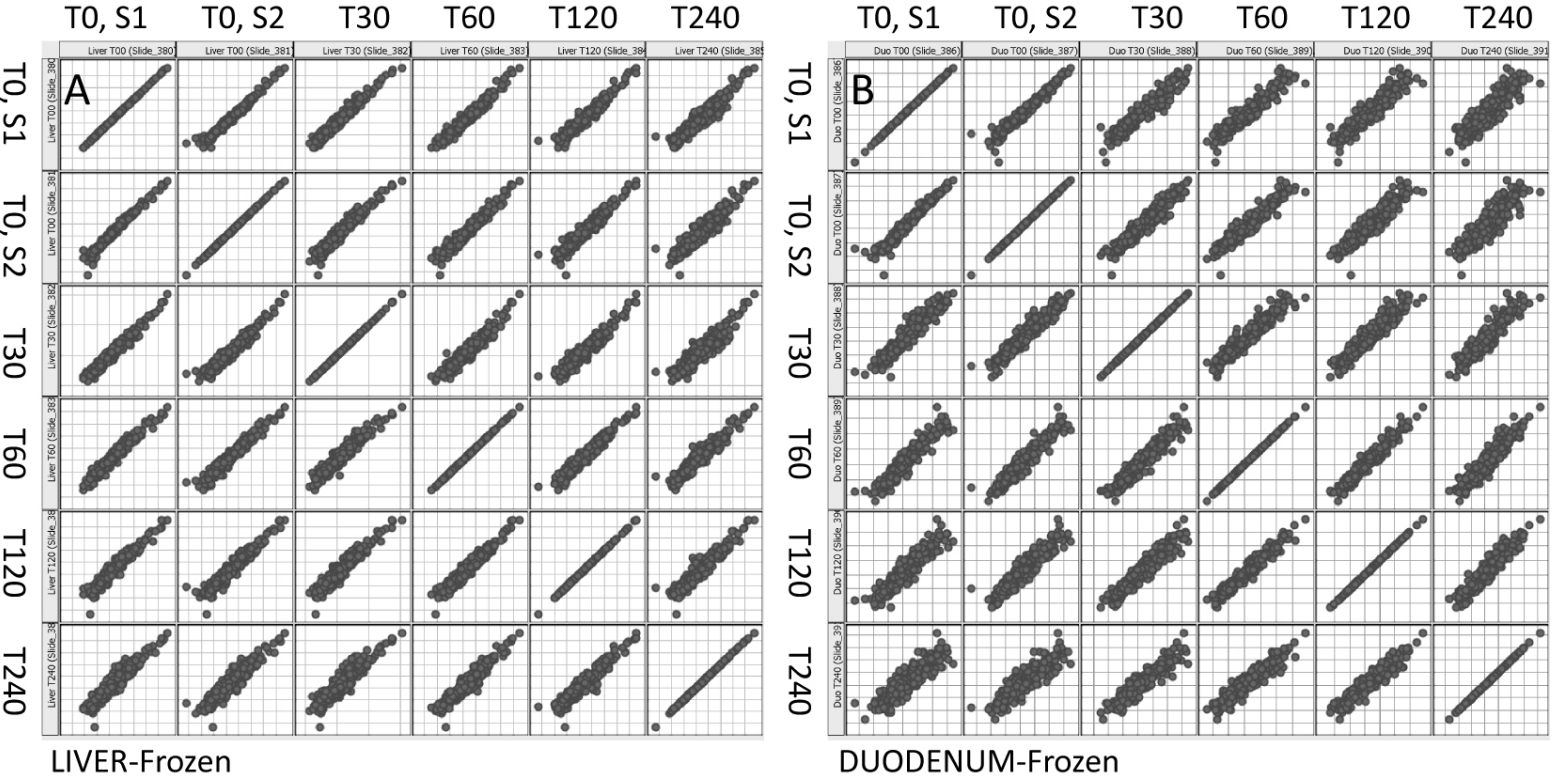
**Scatter plot matrices, snap-frozen tissues**. Matrix plots indicating miRNA expression in snap-frozen samples. Comparison of samples with high versus low RINs for both liver (A) and duodenum (B) show that severe total RNA degradation increases the proportion of miRNAs classified as significantly regulated.

**Figure 5:**
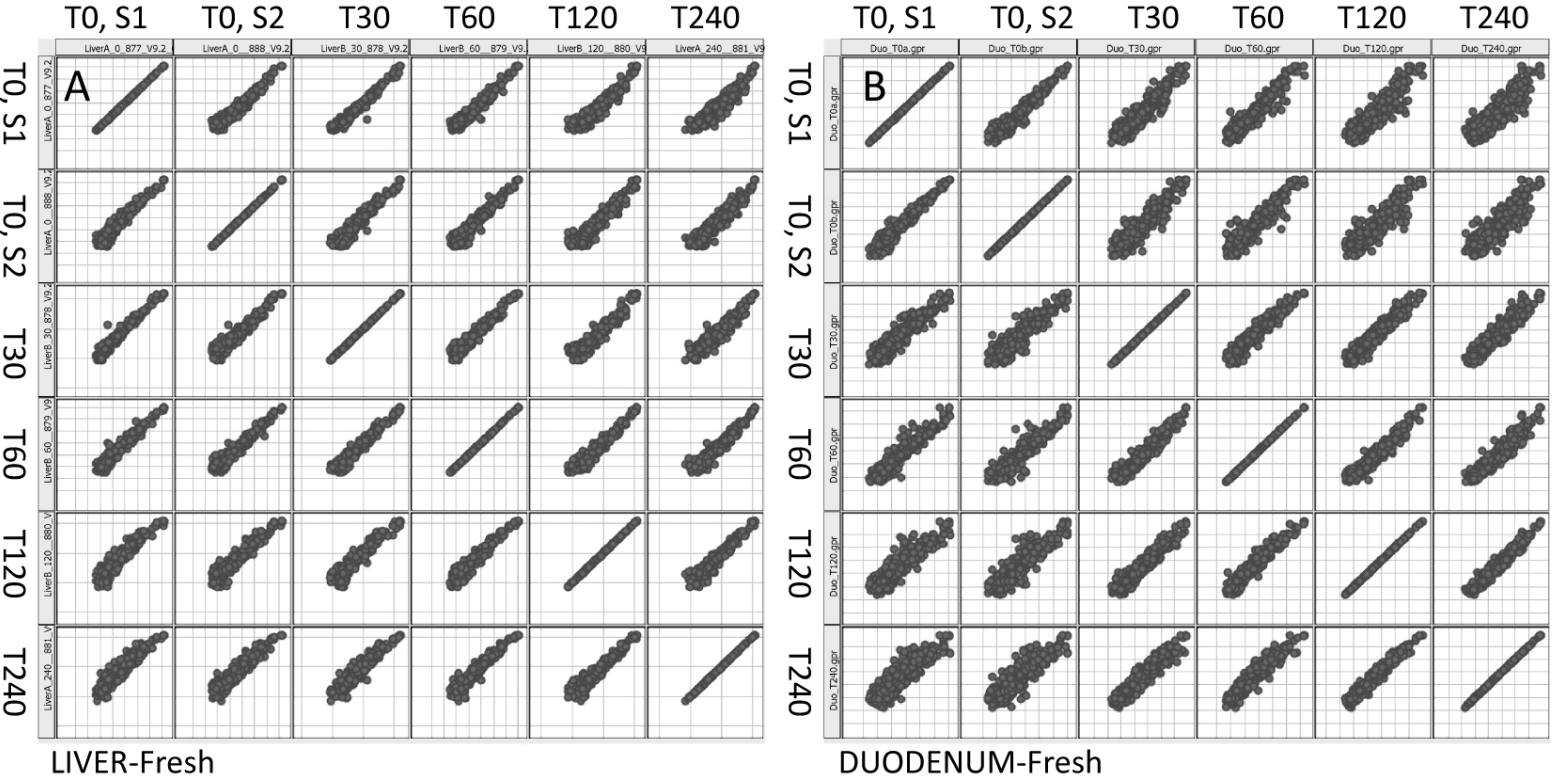
**Scatter plot matrices, freshly harvested tissues**. Matrix plots indicating miRNA expression in freshly prepared tissues. MiRNA expression in freshly prepared liver tissue (A) only show few capture probes that indicate regulation. By contrast, miRNA expression in freshly prepared duodenal tissue (B) is prone to major degradation, therefore, a large number of capture probes indicate significant regulation.

HCL is a robust, global, analytical technique that clusters expression profiles but does not reflect changes in expression of individual miRNAs. To evaluate changes in expression of individual miRNAs we organized the data into scatter plot matrices (Figure 4). A scatter plot matrix presents multiple variables by pair-wise evaluation. Comparison of samples with high *versus *low RINs (e.g. liver T0 RIN, 7.2 *vs *T120 RIN, 2.8; Figure 4A) shows that samples with degraded total RNA increase the proportion of miRNAs classified as significantly regulated. This is also true when comparing degraded samples with similarly low RINs (i.e. duodenum T120 RIN, 2.7 *vs *T240 RIN, 2.5; Figure 4B) which generate a significant number of falsely positive regulated miRNAs. For comparison we have hybridized to miCHIP RNAs extracted from freshly harvested livers and duodenums (Figure 5). Our data show that miRNAs extracted from freshly harvested liver is less degraded, as evident from the matrix plot analyses (Figure 5A). Consistent with high level of RNases present in duodenum, miRNAs from freshly harvested duodenum are extensively degraded (Figure 5B). Based on these data, we conclude that samples with low RIN values (less than 7) do not merit analysis on miRNA arrays.

### Degradation of total RNA randomly affects miRNAs

**Figure 6:**
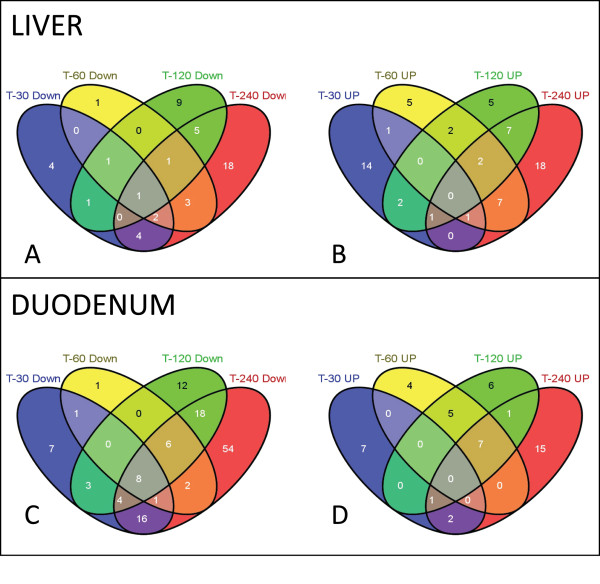
**Venn diagram**. Venn diagrams were used to identify capture probes systematically regulated by total RNA degradation. Lists of significantly regulated capture probes for the defrosted liver (A) and the duodenum (B) were created and the intersection between the lists was assessed by using the Venn diagram. For complete probe list see Additional file 1 - Table S1.

Next, we assessed if total RNA degradation systematically affects miRNA profiles and if so, to devise methods to correct for systematic errors. For this purpose we filtered scatter plot matrices (see Figure 4) and selected those capture probes which show regulation by at least two fold in any of the scatter plots (for the complete list of probes see Additional file 1 - Table S1). To identify whether any capture probe was systematically affected by the degradation, we calculated the overlap between the probe lists (Figure 6). Our analysis shows that, within each tissue (i.e. liver and duodenum) we could not identify over-represented capture probes, indicating that under our experimental conditions miRNA degradation occur randomly and therefore cannot be systematically corrected.

### miRNA analysis by qPCR

**Figure 7:**
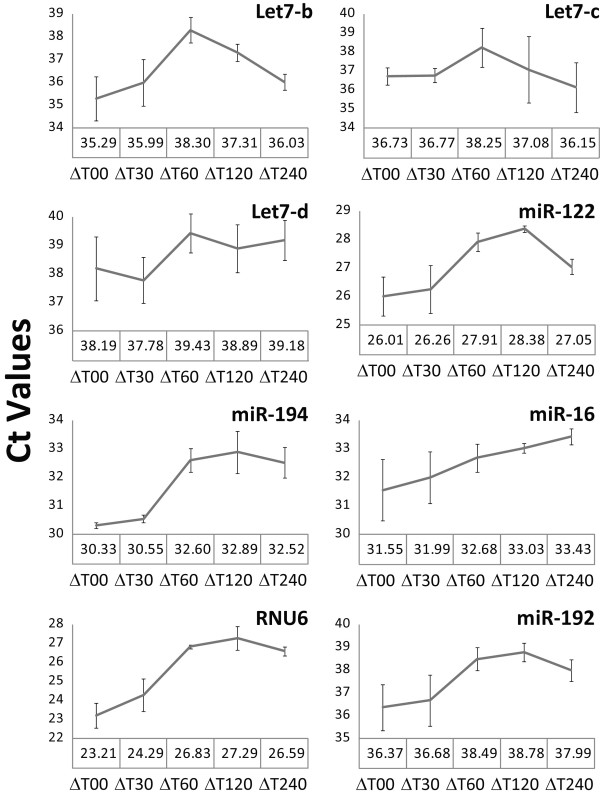
**Effect of total RNA degradation on miQPCR data**. Comparison of changes in relative expression for 7 selected miRNAs (Let-7b, Let-7c, Let-7d, miR-122, miR-16, miR-192 and miR-194) and RNU6 in total RNA extracted from frozen liver. For individual miRNAs the crossing threshold (C_t_) value across the seven different miRNAs is shown. Data are shown as average of three independent miQPCR runs ± standard deviation.

We next tested whether degradation of total RNA also affects expression profiles of miRNAs by using miRNA specific qPCR. For this purpose, we synthesized cDNA from hepatic total RNA (T0, T30, T60, T120 and T240) and analyzed it by the miQPCR method (manuscript in preparation). The abundance of seven liver-expressed miRNAs (Let-7b, Let-7c, Let-7d, miR-122, miR-16, miR-192 and miR-194) and of RNU6 was analyzed by using SYBR Green and the crossing threshold value (C_t _[22]) determined for each condition (Figure 7). These analyses indicate that despite the fact that miRNAs have short amplicons the relative amount that we can detect by miQPCR is compromised by RNA degradation. Different miRNAs are compromised to different extents within the same RNA sample, again indicating that degradation is random and cannot be corrected by post-analytical processing.

## Discussion

Correlation of miRNA expression patterns with clinically relevant information is greatly facilitated by the retrospective analysis of samples archived in tissue banks. Unfortunately, the quality of samples stored in tissue banks is variable due to heterogeneity in pre-analytical preparation of clinical specimens. Collectively, these variables will impact the reliability of the results of the analysis. While total RNA extracted from FFPE blocks is severely degraded, several studies suggest that the miRNA profiles are not significantly compromised despite total RNA degradation arisen from formalin fixation [14-16]. These reports strengthen the commonly held misconception that the short size of mature miRNAs allows them to escape degradation.

To date no systematic studies have been performed to investigate the relationship between total RNA degradation and miRNA profiles determined from snap-frozen tissue collections. To investigate this question, we compared miRNA expression profiles generated through delaying the extraction of RNA from liver and duodenum, to generate different RNA integrities as defined by RIN values. Northern blot analysis shows that the amount of miR-122 decreases in those samples with lower RIN values (Figure 2). In addition, we observe that signal intensities determined by miCHIP microarray are significantly affected when total RNA degradation is observed (Figure 4, 5, 6 and 7). Correlation of the RIN numbers (Figure 1) with the number of "degradation-regulated" capture probes (See Additional file 1 - Table S1) show that miRNAs in the duodenal tissue are affected more severely by degradation compared to miRNAs in the liver.

Previous studies indicate that low RNA integrity still permits the use of short amplicons to reliably monitor mRNA expression by qPCR [23]. By contrast, our data clearly demonstrate that this does not apply to miRNA expression profiling by qPCR. We show that extracted RNA samples that are highly degraded, as characterized by low RINs, containing between two to eight fold less miRNA than compared to the control (T0) RNA (Figure 7).

Collectively, our findings demonstrate that total RNA degradation affects miRNAs as detected by northern blot analysis, array-based miRNA profiling and by miRNA-specific qPCR. Inclusion of samples with degraded total RNA in miRNA expression profiling experiments reduces the analytical power of the analysis through increasing the variation within the experiment, rendering it more difficult to detect biologically significant miRNA signatures reliably.

## Conclusion

We conclude that there is no suitable methodology for the reliable and reproducible analysis of miRNA expression of poor quality RNA samples. In order that miRNA expression profiles reflect true biological differences and not variation imposed by RNA integrity, we propose that only total RNA with integrity equal to, or higher than RIN seven should be included for miRNA expression analysis.

## Methods

### Study design and RNA preparation

Snap-frozen livers and duodenum collected from female mice of Bl6/57 genetic background were stored in the -80°C for more than 12 months. In order to obtain RNA of different quality frozen tissues were cut in five fragments of similar size, placed into individual eppendorf tubes and stored in dry ice. At time point zero (T0) samples were moved into ice and at different time points 1 ml of Trizol (Invitrogen) was added to the sample. Tissues were immediately disrupted by using mechanical shearing (Tissuelyzer, Qiagen). Total RNA containing small RNA was extracted from the homogenate as previously described [20,21]. RNA concentration and RNA quality were assessed by NanoDrop ND100 (NanoDrop Technologies) and Agilent 2100 Bioanalyzer (Agilent Technologies). For comparison, we harvested livers and duodenums from Bl6/57 female mice. Freshly collected tissues were transferred in ice and processed as described above.

### Northern blotting

Northern blot analysis was performed as described [21], 10 μg of total RNA were loaded onto denaturing Poly Acrylamide Gel Electrophoresis (PAGE) and run alongside an appropriate DNA marker. Following electrophoresis, RNA was transferred to nylon membrane using semidry transfer (Semiphor; Pharmacia Biotech). Membranes were then hybridized overnight at 50°C in Phosphate hybridization buffer, together with LNA oligonucleotides (miRCURY LNA Array detection probes; Exiqon) that were previously labeled with P^32 ^by using PNK (Poly Nucleotide Kinase, NEB). Signal intensities were recorded by using phosphoimager (Toshiba) and normalized by using non-saturated images of ethidium bromide stained gel (Gel Analyzer, Biorad) taken before transfer to the membrane. Signal intensities were calculated using ImageJ [24] and plotted using Excel.

### Microarray hybridization and data analysis

Total RNA (2 μg) was labeled and hybridized to miCHIP as previously described [20,21]. miCHIP is based on Tm-normalized capture probes (miRCURY; Exiqon, Denmark). miRCURY probes spotted on these arrays were designed to target 500 (miRBase v9.2 [25]) unique mouse miRNAs. Array images were generated by using the Genepix 4200AL laser scanner (Molecular Devices), miCHIP arrays were scanned in batches using the Genepix auto Photo Multiplayer (PMT) algorithm, with pixel saturation tolerance set to 0.2%. Tiff images generated by the Genepix 4200AL laser scanner were processed by the Genepix 6 microarray analysis software (Molecular Devices). Artifact-associated spots were eliminated both by software- and visual-guided flags. Signal intensities were measured according to the local background subtraction method as a function of the median of foreground pixels minus median of background pixels. Genepix outputs files are imported directly in Genespring and data are normalized to the 50^th ^percentile. Genespring build-in tools were used to visualize box plots, scatter plots and hierarchical clustering. Any additional analysis of the data is done by using Excel and TIGR MeV (MultiExperiment Viewer, http://www.tm4.org[26]). To investigate the relationship between the affected capture probes we have used Venn diagrams. Venn diagrams were plotted by using an online freely available tool named Venny http://bioinfogp.cnb.csic.es/tools/venny/index.html.

### miQPCR

For miQPCR 500 ng of total RNA were reverse transcribed as described (miQPCR is covered by patent number EP 09 002 587.5) and 5 ng of cDNA were used for individual assays. cDNAs were amplified by using gene specific primers and SYBR Green [Applied Biosystems (ABI), P/N 4309155] and run on a ABI 7500 instrument following standard 7500 mode (96-well plate at 50°C for 2 min, 95°C for 10 min, followed by 40 cycles of 95°C for 15 sec, 60°C for 1 min; all reactions were run in duplicates) with dissociation step (ramping from 60°C to 95°C) for monitoring melting curve of the PCR products. After the run threshold cycles (C_t_) were defined as the fractional cycle number at which the fluorescence passed the fixed threshold[22]. C_t _values were extracted by using the Prism7000 software (ABI) and plotted using Excel.

### Microarray repository

Arrays results generated in this study have been deposited in the Gene Expression Omnibus (GEO) repository and are accessible under accession number GSE18788 and GSE19104.

## Competing interests

DI, VB, MUM and MC declare no competing financial interests.

## Authors' contributions

DI performed RNA extraction and microarray experiment; VB and MUM contributed to research and to the writing of the manuscript; MC performed miQPCR, northern blot, analyzed the data and wrote the manuscript. All authors read and approved the final manuscript.

## Supplementary Material

Additional file 1 **Table S1 - Detailed list of degradation-regulated probes as detected by microarray**. The Table contains the list of individual probes identified as regulated (i.e. for displaying fold changes of ± 2) in the microarray experiments. Data sets were correlated as indicate in the legend of Figure 6. Click here for file
